# Autophagy and Neurodegeneration: Insights from a Cultured Cell Model of ALS

**DOI:** 10.3390/cells4030354

**Published:** 2015-08-06

**Authors:** Francesca Navone, Paola Genevini, Nica Borgese

**Affiliations:** Institute of Neuroscience, Consiglio Nazionale delle Ricerche, and Department of Medical Biotechnology and Translational Medicine (BIOMETRA), Università degli Studi di Milano, Via Vanvitelli 32, 20129 Milano, Italy; E-Mails: p.genevini@in.cnr.it (P.G.); n.borgese@in.cnr.it (N.B.)

**Keywords:** autophagy, UPS, protein degradation, autophagy receptors, proteostasis, cytoplasmic inclusions, aggregates, neurodegeneration, ALS, VAPB

## Abstract

Autophagy plays a major role in the elimination of cellular waste components, the renewal of intracellular proteins and the prevention of the build-up of redundant or defective material. It is fundamental for the maintenance of homeostasis and especially important in post-mitotic neuronal cells, which, without competent autophagy, accumulate protein aggregates and degenerate. Many neurodegenerative diseases are associated with defective autophagy; however, whether altered protein turnover or accumulation of misfolded, aggregate-prone proteins is the primary insult in neurodegeneration has long been a matter of debate. Amyotrophic lateral sclerosis (ALS) is a fatal disease characterized by selective degeneration of motor neurons. Most of the ALS cases occur in sporadic forms (SALS), while 10%–15% of the cases have a positive familial history (FALS). The accumulation in the cell of misfolded/abnormal proteins is a hallmark of both SALS and FALS, and altered protein degradation due to autophagy dysregulation has been proposed to contribute to ALS pathogenesis. In this review, we focus on the main molecular features of autophagy to provide a framework for discussion of our recent findings about the role in disease pathogenesis of the ALS-linked form of the *VAPB* gene product, a mutant protein that drives the generation of unusual cytoplasmic inclusions.

## 1. Autophagy: Main Features and Links with the Ubiquitin-Proteasome System

Cellular homeostasis requires a constant balance between biosynthetic and catabolic processes. In particular, protein turnover is essential, both for maintaining the pool of amino acids required for continued protein synthesis and for removing defective proteins that are translated or fold incorrectly. Furthermore, many essential cellular functions, such as cell division, transcription and signal transduction, are regulated by the modulation of protein levels accomplished by altering the balance of protein synthesis and degradation [1].

The two major intracellular protein degradation pathways are: the ubiquitin proteasome system (UPS) and autophagy. The term “autophagy” refers to a range of processes, including chaperone-mediated autophagy, microautophagy and macroautophagy, the latter being the major and best characterized subtype of autophagy and the one we refer to as autophagy in the present review [2,3,4,5].

**Figure 1:**
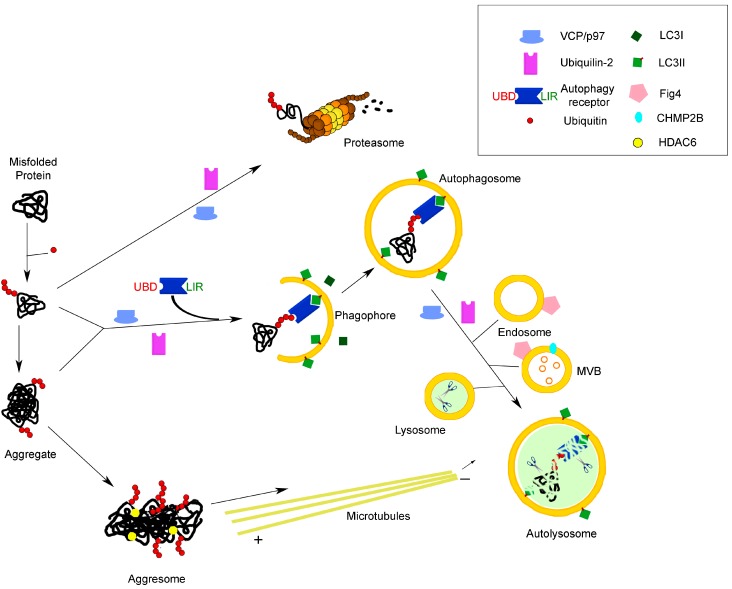
Molecular links between the two main protein degradation pathways in the cell: the proteasomal and autophagolysosomal systems. The cartoon illustrates the involvement of receptors/adaptors/chaperones common to both systems in the process of the degradation of misfolded proteins, as an example of a function highly relevant to neurodegenerative diseases, such as ALS. See the text for further explanation. UBD, ubiquitin-binding domain; LIR, LC3-interacting region; MVB, multivesicular body.

The UPS selectively eliminates many short-lived proteins and confers the specificity and temporal control needed for fine-tuning the steady-state levels of normal regulatory proteins. Its function is also responsible for the degradation of abnormally-folded or damaged proteins that arise by missense or nonsense mutations, biosynthetic errors or damage by oxygen radicals and is important for antigen presentation in immune surveillance. Moreover, it serves to maintain amino acid pools in acute starvation [1]. The UPS is a proteolytic machinery in which a series of sequential and concerted enzymatic reactions leads to the covalent linking of several ubiquitin molecules to proteins, an elaborate ubiquitin-conjugating process that marks them for degradation. Polyubiquitylated proteins are recognized by the proteasome, a very large barrel-shaped multicatalytic protease complex that degrades ubiquitylated proteins to small peptides and guarantees their rapid removal [6,7,8] (Figure 1). Misfolded endoplasmic reticulum (ER) proteins are degraded by the UPS after being retrotranslocated back into the cytosol (a process known as ER-associated degradation (ERAD)).

Autophagy is a highly conserved and tightly-regulated cellular self-degradative process involved both in the basal turnover of cellular components and in the response to chronic nutrient starvation or organelle damage. It involves the degradation of bulk cytoplasm, long-lived proteins, entire organelles, oligomers and protein aggregates through their sequestration by double-membrane vesicles called autophagosomes; these are trafficked to reach lysosomes for fusion or, alternatively, endosomes before fusion with lysosomes, where they are ultimately degraded for subsequent recycling of the products of hydrolysis [3,4,9]. Genetic studies have identified a class of autopha*g*y-related, or Atg, proteins, which are essential for the process and coordinate specific steps in autophagy induction, autophagosome biogenesis and cargo sequestration (see [10,11] for updated reviews on the molecular mechanisms of autophagy). Autophagy has long been considered as a non-selective process responsible for the bulk sequestration of cytoplasmic components. However, whereas the autophagy response to starvation is the bulk degradation of cytosolic material, it is now clear that selective autophagy ensures specific recognition and removal of various cytosolic cargoes, such as aggregated proteins, damaged organelles and pathogens. Thus, numerous types of selective autophagy have been uncovered recently, and under specific conditions, autophagosomes can sequester and degrade in an exclusive way mitochondria (mitophagy), peroxisomes (pexophagy), endoplasmic reticulum (reticulophagy), portions of the nucleus (nucleophagy), cytoplasmic aggregates (aggrephagy), ribosomes (ribophagy), invading pathogens (xenophagy), as well as endosomes, lysosomes, lipid droplets and secretory granules (see [12,13,14,15,16,17] for extensive reviews). Selective autophagy relies on cargo-specific autophagy receptors that facilitate cargo sequestration into autophagosomes (Figure 1).

The UPS and autophagy pathways have long been regarded as two independent proteolytic pathways, serving distinct functions. Nevertheless, connections between these two systems have recently been suggested, and several functional and molecular links have been demonstrated between the two degradative systems, their concerted regulation being fundamental for a balanced proteostasis and normal functioning of all cells, including neurons. For example, UPS and autophagy can be co-activated during endoplasmic reticulum (ER) stress [18], while upregulation of autophagy occurs as a compensatory mechanism when the proteasome capacity is overloaded and its function inhibited by protein complexes resulting from aberrant assembly of misfolded proteins (aggresomes) or protein aggregates [19,20,21,22,23]. The inhibition of autophagy, in turn, has been shown to have a strong impact on UPS activity in different kinds of cells, leading to a decrease of UPS flux and compromising degradation of UPS substrates [13]. An overall regulation and coordination of proteasomal and autophagic degradation at a transcriptional level has been provided by studies showing that transcription factor FoxO3 induces transcription of genes whose products are involved in the upregulation of both proteasomal and autophagic degradation pathways [24,25].

The tremendous progress in the elucidation of the molecular mechanisms underlying autophagy has revealed that the most prevalent autophagy targeting signal in mammals is the modification of cargos with ubiquitin, indicating this cargo signal as a unifying factor of UPS and selective autophagy [13,15,23] (Figure 1). Moreover, the UPS and autophagy lysosomal system use common adaptors, which can direct ubiquitylated target proteins to both degradation pathways.

Ubiquitin acts as a modifier to determine protein fate by covalent attachment to cellular proteins through an enzymatic cascade, which involves three classes of enzymes, termed E1 (activation), E2 (conjugation) and E3 (ligation), generating ubiquitin conjugates that contain either mono- or polyubiquitin chains. Typically, four or more ubiquitin molecules multimerize through conjugation to a lysine residue (K) of another ubiquitin or a substrate protein. While K48-linked polyubiquitin chains are employed by the UPS, substrates recognized by the autophagosome-lysosome pathway are usually modified by K63-linked chains, thus indicating that the structural complexity of the different polyubiquitin chains contributes to the selectivity and specificity of the UPS and autophagy towards their substrates. For example, the same E3 ligase, such as Parkin, can either direct substrates to the proteasome via K48-linked ubiquitylation or form K63 and K27 polyubiquitin chains that are required for efficient degradation of non-functional mitochondria. Ubiquitin chains can undergo remodelling through the action of a large class of deubiquitylating enzymes, proteases that cleave the isopeptide bond between ubiquitin and its substrate [13,15,16,24].

A major role in the regulation and coordination of the UPS and autophagy degradative pathways has been recently demonstrated for autophagic receptor proteins [17,26]. More than 20 autophagy receptors have been identified; however, only a few have been characterized in detail so far, such as, for example, p62/sequestosome 1 (SQSTM1), NBR1, NDP52 and optineurin, which act as cargo receptors for ubiquitylated substrates (Figure 1). These proteins selectively recognize autophagic cargo via their ubiquitin-binding domain (UBD) and mediate its engulfment into autophagosomes by binding to the small ubiquitin-like molecules (Ubl) of the Atg8 family, which comprises the microtubule-associated protein light chain 3 family (LC3 family), the γ-aminobutyric acid (GABA)-receptor associated proteins (GABARAPs) and GABARAP-like proteins subfamilies. The covalent conjugation of Atg8 family proteins to the membrane lipid phosphatidylethanolamine (PtdEth) is essential for them to exert their function in autophagy: lipidation of LC3, the best characterized member of the Atg8 family, determines its conversion from the cytosolic form LC3-I to the autophagosome-associated LC3-II form. The interaction of autophagic receptors with LC3-II is made possible by a short LC3-interacting region (LIR) sequence, which was first identified in p62/SQSTM1 [15,17,27,28]. Specific binding of autophagy receptors to LC3 leads to their recruitment at the inner surface of nascent autophagosomes and promotes the organelle maturation around the intracellular material to be disposed of [4,27,28]. LC3-II is the most widely-used marker to study autophagy, as it specifically associates with both sides of the autophagosome membrane, where it remains bound even after fusion with lysosomes. After the fusion process, LC3-II on the cytosolic face of autophagosomes can be recycled to LC3-I, while the LC3-II on the inner face of the membrane is degraded [28,29]. The conjugation of Atg8/LC3 to and its removal from phosphatidylethanolamine is essential for autophagy, and this process is mediated by an ubiquitylation-like conjugation system functionally and biochemically similar to the UPS (reviewed in [15,27]).

Autophagic receptor proteins, which bind both ubiquitin and autophagy-specific ubiquitin-like molecules (Ubl) like Atg8/LC3, provide the connection between ubiquitylation and autophagy family proteins. These, in turn, contribute to the assembly of molecular platforms on which autophagosomes form [14,15,16,27]. However, although ubiquitylation is frequently a prerequisite for substrate recognition and determines selectivity in autophagy in eukaryotes, other forms of selective autophagy that do not use ubiquitin modifications as a degradation signal have been described [17,30]. For instance, optineurin (see below) can selectively recruit cargoes in an ubiquitin-independent manner to determine autophagic clearance of protein aggregates [17,30].

Autophagy receptors often act as multifunctional adaptor proteins and play key regulatory roles in various biological processes. One such example is represented by p62/SQSTM1, a multi-domain scaffold adaptor protein, which, in addition to regulating the selective autophagosomal degradation of large protein aggregates, mitochondria and bacterial pathogens, is also involved in in a variety of signaling responses to growth factors, neurogenesis, osteoclastogenesis, inflammation, oxidative stress response and apoptosis [14,31,32]. Recently, p62 has been described to play a role in autophagosome formation thanks to its ability to self-oligomerize and form a nucleating scaffold together with polyubiquitylated aggregates, thus giving a major contribution to the formation of inclusion bodies typical of disease states. Given its association with misfolded ubiquitylated proteins in cytoplasmic inclusions and its removal from the cytoplasm mainly by autophagy, its amount is generally considered to inversely correlate with autophagic activity [27,32].

The adaptor protein NBR1 is a major autophagy receptor for peroxisomes, which, through its own UBD domain and LIR motif, can participate in the recruitment and autophagosomal degradation of ubiquitylated proteins. It is an interaction partner of p62 and can be found located at inclusion bodies associated with human pathologies. Like p62, NBR1 is itself a substrate for selective autophagy [14].

Optineurin has emerged as a key player in the regulation of various physiological processes through its ability to interact with numerous binding partners, including LC3. Optineurin is an inhibitor of nuclear factor-κB (NF-κB) activation and is involved in transcriptional regulation, as well as cell division and immune response. Additionally, optineurin plays an important role in the maintenance of the Golgi complex, membrane trafficking and exocytosis, through its interaction with myosin VI and Rab8. Its role in selective autophagy is complex and essential to achieve an efficient autophagic clearance of protein aggregates via both an ubiquitin-dependent and -independent pathway (reviewed in [33,34]). Recently, optineurin has been reported to participate in the autophagic degradation of damaged mitochondria [35] and to be able to functionally interact with p62/SQSTM1 to form an autophagy receptor complex that accelerates autophagic flux for the suppression of tumor growth [36]. Optineurin and NDP52 have been described to act as xenophagy receptors, since they bind ubiquitylated intracellular pathogens and LC3 simultaneously and utilize the autophagic machinery for restriction of bacterial infections, such as that of *Salmonella enterica* (reviewed in [28,33]).

Another adaptor protein capable of binding polyubiquitylated substrates is ubiquilin-2 (UBQLN2), also a member of the ubiquitin-like protein family, which recognizes both the proteasome complex and (although probably indirectly) LC3, thus delivering ubiquitin-tagged proteins to both proteasomal and autophagic degradation [37]. Notably, ubiquilin-2 works in concert with other autophagy-related proteins, such as optineurin, to regulate the constitution of endosomal vesicles [38] and the ATP-driven chaperone valosin-containing protein (VCP)/p97 to mediate ER-associated degradation [39].

The valosin-containing protein VCP/p97, also called cdc 48, belongs to the exameric AAA (ATPase associated with diverse cellular activities) family of proteins and can convert the energy of ATP hydrolysis to structurally remodel or unfold client proteins. Mutations that abrogate ATP binding or hydrolysis result in dominant negative variants that bind, but cannot release substrates, thus inducing the accumulation of ubiquitin conjugates. Although VCP/p97 cannot be technically considered an autophagy receptor, because no direct interaction has been shown for this protein with LC3 proteins, it plays a central role in protein quality control to protect cells from the cytotoxic effects of misfolded and aggregation-prone proteins. p97 binds to ubiquitylated substrates with the help of a network of interaction partners and protein cofactors and, by catalyzing remodelling of client structures, triggers their extraction from complexes or cellular membranes, thus regulating a wide range of cellular functions (reviewed in [40,41]). For example, as a central element of the UPS, it plays a key role in ERAD and is essential for extraction and delivery to the proteasome of both ER luminal and membrane proteins. In addition to substrate extraction, p97 has been proposed to assist the proteasome in unfolding proteins to facilitate their degradation and to regulate proteasome-mediated degradation of outer mitochondrial membrane proteins. VCP/p97 is also involved in proteasome-independent degradation in the lysosomal and autophagolysosomal systems through controlling protein sorting in the endocytic pathway and endolysosomal trafficking [40,41]. The activity of VCP/p97 in the ubiquitin system is also important in key intracellular signaling pathways, cell cycle regulation, as well as chromatin-associated functions relevant to DNA repair and genome stability. Finally, VCP/p97 has been recently shown to play a major role in RNA metabolism and autophagy-mediated clearance of mRNA-containing stress granules, which are commonly observed in neurons of patients with neurodegenerative diseases and are composed of cytoplasmic mRNA and RNA-binding proteins [42], reviewed in [41].

The enzyme microtubule-associated histone deacetylase 6 (HDAC6) is an additional molecular link between the proteasomal and autophagic machinery. When the proteasome is inhibited by an overwhelming load of misfolded proteins, aggresome formation occurs, as a specific cellular response to create repositories of misfolded protein aggregates [19,43]. HDAC6 was demonstrated to promote aggresome formation and to reduce the toxicity of misfolded proteins, via its ability to interact with polyubiquitylated proteins and dynein motor complexes, thus creating a physical link between ubiquitylated cargo and transport machinery (Figure 1). It mediates microtubule-based transport of protein aggregates to the close proximity of microtubule-organizing centers (MTOC), where lysosomes are enriched, and promotes their degradation by autophagy [21,23,44]. HDAC6 also controls the fusion of autophagosomes with lysosomes by recruiting the machinery that assembles filamentous actin, which, in turn, stimulates autophagosome-lysosome fusion [45]. Interestingly, p97/VCP interacts with HDAC6 and facilitates aggresome formation [23]. Cells lacking HDAC6 activity are unable to use autophagy to compensate for impaired UPS function, and in a *Drosophila* model of neurodegenerative disease, HDAC6 was shown to be essential to rescue neurodegeneration caused by UPS failure via autophagy induction [46].

Although not directly involved in the cross-talk between proteasomal and autophagy-mediated pathways, the multivesicular body protein 2b (CHMP2B) plays an important role in protein degradation. It constitutes a subunit of the endosomal sorting complex required for transport III (the ESCRT-III complex, one of the four multiprotein complexes required for multivesicular body formation) and is involved in the trafficking of ubiquitylated proteins along the endosomal pathway, via multivesicular bodies (MVBs), to lysosomes. As illustrated in Figure 1, fusion of MVBs with autophagosomes is part of the maturation process of these degradative organelles to become autolysosomes; however, it is not clear, yet, whether the role of ESCRT proteins in autophagy is only an indirect one, via lysosome biogenesis, or if they have a direct role in autophagosome closure (reviewed in [10]). Expression of mutant CHMP2B has recently been associated with accumulation of autophagosomes and neurodegeneration [47,48]. Likewise, the lipid phosphatase FIG4, which is located in the endolysosomal membranes and is responsible for the modulation of the phosphoinositide PI(3,5)P2 levels, is implicated in the proper function of the endosomal/lysosomal compartments and, thereby, in autophagy and neurodegeneration [4,12,49,50].

Many proteins can be substrates of both the UPS and autophagy pathways, and the mode of degradation of a misfolded, redundant or unneeded protein may be often governed by the momentary activity or capacity of these cellular quality systems, which are on-going constitutively in most cells at a low level. When proteins are accessible to both the UPS and autophagy pathways, the UPS is the most efficient, favored and dominant clearance route. However, substrates need to be unfolded to pass through the narrow pore of the proteasome barrel, which precludes the clearance of oligomeric or aggregated proteins. When a cytosolic protein is aggregate-prone and a poor proteasome substrate, then autophagy is thought to become the main clearance route and more effective than the proteasome [22,51]. Malfunction of the proteasome and autophagy degradation pathways leads to the accumulation of abnormal, misfolded proteins and damaged components inside cells.

The accumulation of toxic protein aggregates is a hallmark of several common human diseases, from late-onset neurodegenerative disorders to cancer, infections, chronic liver disease, muscle disease and forms of heart failure, so-called protein misfolding disorders or proteinopathies [6,51,52]. Moreover, direct evidence of the connection between *ATG* gene dysfunction and human diseases has recently emerged [53].

## 2. Autophagy and Neurodegeneration

Neurons are long-lived, terminally-differentiated cells that do not undergo renewal. Due to their extreme polarization, size and post-mitotic nature, they are uniquely sensitive to the accumulation of misfolded proteins, dysfunctional organelles and protein aggregates, because they cannot rely on the dilution of cellular waste occurring during cell division (see [11,54,55] for reviews).

In agreement with the morphological and functional specialization of neurons, autophagosome biogenesis and maturation are spatially and temporally regulated in neuronal cells: after forming preferentially at the axon tips, where ubiquitylated protein aggregates bind different LC3-II-interacting cargo receptors, they are transported by dynein motors along the axon towards the cell soma [56]. During the transport, autophagosomes fuse with multivesicular bodies and late endosomes, forming amphisomes, and these, finally, fuse with lysosomes to create autolysosomes. Fusion of autophagosomes with lysosomes initiates the degradation of autophagic cargo, and all pathological situations that interfere with these maturation processes lead to the accumulation of autophagosomes in axonal swellings ([11,56,57] and the references therein).

In neurons, constitutive (basal) autophagy is relatively active and extremely important for the continuous turnover of cytosolic proteins and the maintenance of normal cellular homeostasis; indeed, autophagy is so efficient and rapid, that it is barely detectable in healthy neurons, where low levels of autophagosomes can be detected. The protective function of basal autophagy against the onset of neurodegeneration was originally demonstrated by genetic studies in which selective knockout of essential autophagy genes Atg5 or Atg7 in neuronal cells causes both a phenotype closely resembling those seen in neurodegenerative diseases and aggregation of ubiquitylated proteins in the cytoplasm, even in the absence of disease-associated mutant proteins [58,59]. Interestingly, autophagy deficiency results in specific progressive degeneration of axon terminals, leading to axonal dystrophy, suggesting that axons are much more vulnerable to autophagy defects than dendrites [54,60]. The pathological response induced by the blockade of autophagy cannot be rescued by the upregulation of the UPS system [46]. This explains the large number of pathologies resulting from poor quality control in neurons, especially as the brain ages (*i.e.*, neurodegenerative diseases) [11,61,62].

Beyond its role in the clearance of misfolded proteins spontaneously generated during routine protein turnover, autophagy plays an important role in the clearance of aggregate-prone mutant proteins associated with several different neurodegenerative diseases [51]. In this respect, the accumulation of autophagic vesicles often observed in these disorders has raised the question of whether it may represent an increase in autophagy or a block in the removal of autophagosomal vacuoles, often leading to a controversial interpretation of autophagy function in neurodegenerative diseases (reviewed in [63]). In these pathological conditions, which share the common feature of accumulation of misfolded proteins, the specific protein, cell type and cellular localization of such accumulations vary among the diseases. For example, Parkinson’s disease is characterized by the presence of cytoplasmic aggregates of mutant α-synuclein (Lewy bodies), whereas in the polyglutamine expansion disorders, aggregates are seen either predominantly within the cytoplasm, as in Huntington’s disease, or in the nucleus, as in the case of spinocerebellar ataxia type I. In Alzheimer’s disease, both intracellular tau aggregates and extracellular β-amyloid aggregates are seen. Another group of proteinopathies of the nervous system is represented by motor neuron diseases (MND), a spectrum of neurodegenerative syndromes affecting selectively motor neurons, which includes ALS and is characterized by the presence of aggregates of different mutant proteins, such as TDP43 (see below). Other protein species in the aggregate may not be mutated. Since the proteasome is unable to degrade oligomeric species of these aggregate-prone proteins, a shift towards autophagic degradation ensues and, in parallel, the inhibition of ERAD function and induction of ER stress [51,64].

Although autophagosomes are able to engulf oligomeric species and small aggregates, they may clear large inclusion bodies with more difficulty. Whether these inclusion bodies are toxic, through a variety of suggested mechanisms, or, rather, protective because of their capacity to sequester toxic oligomeric species, is still a matter of debate, and either of the two alternatives may predominate in different situations. Remarkably, recent advances using induced-pluripotent-stem-cell (iPSC)-derived motor neurons from ALS patients carrying SOD1 mutations have recapitulated essential disease features in the absence of mutant SOD1 aggregation, consistent with the idea that soluble mutant proteins can have substantial phenotypic effects ([65,66,67], reviewed in [68]).

According to one view, inclusion body formation is an event secondary to a failure to control protein turnover in the absence of a properly functioning autophagic activity [51,52,54,69]. In another view, large deposits of misfolded mutant proteins may on their own disturb autophagic function both by acting via a gain of toxic function and by sequestering functional proteins important for the autophagy process; in this case, a loss-of-function of autophagy would be a secondary pathological mechanism [52,70]. Reconciling these two views on autophagy failure as primary cause or secondary phenomenon, it appears in any case that a vicious cycle between protein aggregates and autophagy is established in cells destined to degenerate. Upon progression of the disease state, both proteasome activity and autophagy finally decline, contributing to the increase in the overload of misfolded proteins, generation of chronic ER stress and cell death [22,51,64,71].

The high relevance to neuronal health of a correctly balanced function of protein and organelle degradation pathways is further strengthened by recent research linking mutations in autophagy-related genes to inherited neurodegenerative diseases. For example, there has been a great deal of interest in the *PINK1* and *PARK2* genes, which code for a mitochondrial ser/thr protein kinase and an E3 ubiquitin ligase, respectively. These proteins are key players in the selective degradation of damaged mitochondria by autophagy (mitophagy) and are linked to hereditary forms of Parkinson’s disease (reviewed in [72]). Similarly, as will be discussed in the next chapter, a number of genes, coding for cargo receptors or involved in pathways associated with autophagosome trafficking and maturation, have been linked to hereditary forms of ALS.

## 3. Autophagy in the Pathogenesis of ALS

ALS is a complex and heterogeneous disease, the most frequent adult-onset MND that affects both lower motor neurons in brain stem and spinal cord and the upper motor neurons in the motor cortex. Loss of these neurons leads to muscle atrophy and weakness, resulting in progressive paralysis of voluntary muscles. ALS is one of the most rapidly progressive and lethal neurological disorders, usually fatal within five years of onset. Cognitive dysfunction is detected in about one third of patients with ALS, as a result of the involvement of other neuronal populations in the frontal and temporal regions of the cortex, and fronto-temporal lobar degeneration (FTLD) is present in about 10% of ALS patients, so that the two clinical conditions are thought to be at the opposite ends of a same spectrum of disorders [73].

Due to its untreatable nature and fatal evolution, ALS appears as a rare disease (prevalence of 4–6 per 100,000 each year), although its incidence is of 1–2 per 100,000 each year. At present, no effective therapy appears to modify the course of this terrible disease, and predictive diagnostics are limited and insufficient [74,75].

Most cases (90%) of ALS are classified as sporadic (SALS), as they are not associated with a documented family history. The remaining 5%–10% are familial (FALS) and usually of autosomal dominant inheritance. A landmark discovery in ALS research was the finding that mutations in the gene encoding the enzyme superoxide dismutase SOD1 are responsible for 20% of inherited ALS cases, where normal SOD1 is a crucial intracellular antioxidant, facilitating the clearance of the potentially toxic superoxide radicals and protecting cells from reactive oxygen species (ROS)-mediated damage, such as the formation of toxic oligomers and protein aggregates from misfolded proteins [76]. Since then, several other genes have been linked to FALS. Mutations in the RNA/DNA-binding proteins TDP-43 and FUS cause a small fraction (~5%) of FALS each. Hexanucleotide repeat expansions in *C9ORF72* are the most common (40%–50%) genetic cause of ALS, and their identification meant a leap forward in the field of ALS genetics, because it established the molecular link between ALS and FTLD. Several other genes have been identified as genetic causes of ALS, and collectively today, over 60% of FALS can be explained by known mutations. While the etiology of the vast majority of SALS remains unknown, mutations in genes known to cause FALS were identified in a small fraction of apparently sporadic patients, thus revealing an unexpected clinico-pathological link between SALS and FALS. For example, intronic hexanucleotide repeat expansions in *C9ORF72* cause approximately 7% of SALS without familial history [77]. The clinical and pathological similarity between sporadic and familial forms suggests at least some common pathogenetic pathways. Hence, unraveling the disease mechanisms of FALS-linked genes is important to understand the pathogenesis of the much more frequent sporadic forms.

The study of model systems of the genetic forms of ALS has led to major advancements towards unraveling the molecular pathways underlying the complex pathology of ALS. Multiple pathogenic mechanisms have been shown to contribute to motor neuron injury not only in the best characterized SOD1 mutation [76], but also in several of the above-mentioned ones: from oxidative stress, mitochondrial dysfunction and excitotoxicity to dysregulated transcription and RNA processing, ER stress, altered endosomal trafficking and neuroinflammation, the convergence of damage developed within multiple cell types, including non-neuronal supporting cells [78], it appears to be crucial to neuronal dysfunction (see [74,79] for extensive reviews). However, how these factors are temporally and causally linked to each other and to motor neuronal death is unclear.

Among the initiating events by which mutant proteins may induce ALS, a substantial body of evidence points to a failure of proteostasis. Like in other neurodegenerative diseases, the pathological hallmark of ALS is the presence of ubiquitin-positive inclusions consisting of misfolded protein aggregates in the affected motor neurons and glial cells of the spinal cord and motor cortex [80]. Several studies have identified mutant SOD1, FUS, TDP-43 and the translational product of intronic repeats in the gene *C9ORF72* as constituents of ALS-linked cellular aggregates. The current view is that these aggregated structures or, more likely, their oligomeric precursors, directly exert neurotoxic effects by disturbing normal homeostasis and inducing cellular stress [75]. Large aggregates, in turn, could contribute to this toxic role either by causing “traffic jams” and inducing axonal transport impairment, mitochondria disorganization/dysfunction and sequestering functional proteins or by interfering with the UPS and the autophagic degradative pathway. Indeed, mutant SOD1, TDP43, FUS and C9ORF72 have all been shown to inhibit the autophagic flux and lead to clearance deficits (reviewed in [11,81]). As in the case of other neurodegenerative diseases, the boundary between a possible primary and/or secondary pathogenic role of aggregates is therefore quite a blurred one.

An important link between protein aggregates and altered RNA metabolism has emerged since the identification and characterization of ALS-causing mutations in the DNA- and RNA-binding proteins TDP-43 and FUS. These multifunctional proteins, both major constituents of ubiquitin inclusions that neuropathologically characterize ALS, normally reside in the nucleus and are implicated in transcription, pre-mRNA splicing, RNA transport and microRNA biogenesis (reviewed in [34,75]). TDP-43 and FUS shuttle between the nucleus and the cytoplasm and associate with granules in which RNA is translationally silenced and transported to target sites, such as the postsynaptic compartment of neurons, where translation is resumed. While this process is reversible in physiological conditions, or when an acute and intermittent stress is removed, resulting in the dissolution of granules and relocation of the proteins to the cell nucleus, chronic stress, as well as pathogenic mutations favor the recruitment and irreversible accumulation of TDP-43 and FUS into stress granules. These are believed to constitute a pro-aggregation environment and to facilitate the formation of inclusions typical of ALS, where other key mediators of RNA processing and RNAs can be sequestered, thus triggering large-scale deficits in RNA metabolism. The presence of a prion-like domain in the TDP-43 and FUS proteins is necessary for stress granule formation and may also be important for the propagation of neurodegeneration in a RNA-dependent manner (reviewed in [75,77,82]). Autophagy controls the turnover of stress granule-based aggregates, and inhibition of autophagy, lysosome and VCP/p97 function has been reported to impair stress granule assembly (Seguin S.J. *et al*., 2014). Moreover, the clearance of stress granules is also dependent on VCP-mediated autophagy [42]. Of note, other ALS-FTLD-linked genes are associated with stress granule function, including hexanucleotide repeat expansion containing mutant *C9ORF72*, which accumulates in nuclear RNA foci, sequestering essential nuclear RNA-binding proteins and producing splicing dysregulation correlated with ALS disease severity [42,75,83].

The involvement of defective autophagy as a triggering event in the etiology of ALS-FTLD was first suggested by the increased numbers of autophagosomes in the cytoplasm of motor neurons of sporadic ALS patients, thus suggesting that the process is activated and upregulated in this disease [84], reviewed in [85]. The strongest and most recent evidence that disturbances of the autophagic process may be central to in the pathophysiology of ALS comes from the demonstration that a number of FALS-linked mutations affect proteins that are directly involved in proteostasis and, more specifically, represent autophagy receptors and regulators [5,34,55,75]. Recent studies have also highlighted a functional interplay between autophagy and RNA processing, supporting an integrated model that potentially brings together several ALS-FTLD-associated genes into a common pathway [34].

An updated and complete overview of the ALS-associated genes and mutant proteins possibly implicated in altered mechanisms of autophagy has been recently provided [34]. Although it is not the scope of this review to discuss in detail the ALS-linked mutant molecules and their implications in the pathogenesis of the disease, below we provide a schematic summary of the ones associated with defective autophagy (Table 1), highlight their role in the autophagy process and correlate them with the reported phenotype.

**Table 1:** Genetic causes of ALS associated with defective autophagy.

Mutant Molecule	Gene Locus	Inheritance	Predominant Phenotype	Autophagy Defect	Estimated %	Reference
SQSTH1/p62 (sequestosome)	5q35	Dominant	ALS/ALS + FTLD	Substrate recognition Selective autophagy	Unknown	[86] Reviewed in [32]
Optineurin	10p15-p14	Dominant: missense mutations; Recessive: Exon 5 deletions and nonsense mutations	ALS/ALS + FTLD	Autophagosome recruitment to damaged mitochondria	1%–4%	[35,87]
Ubiquilin-2	Xp11.21	X-linked dominant	ALS/ALS + FTLD	Substrate recognition and selective autophagy	<1%	[88] Reviewed in [89]
VCP/p97 (valosin-containing protein)	9p13.3	Dominant	ALS/ALS + FTLD	Autophagosome maturation	1%–2%	[90]
Dynein/Dynactin	2p13	Dominant	ALS	Autophagosome transport and fusion with lysosome	Unknown	[91]
CHMP2B (Charged Multivesicular body protein 2b)	3p11	Dominant	ALS/ALS + FTLD	Autolysosome formation	Unknown	[47,92]
FIG4 (Phosphatidylinositol 3,5-bisphosphate 5-phosphatase)	6q21	Dominant	ALS	Alteration of endo-lysosomal trafficking	Unknown	[93]
ALS2 (Alsin)	2q33.2	Recessive	ALS	Alteration of endosomal trafficking	<1%	[94]
C9ORF72	9p21.3-p13.3	Dominant	ALS + FTLD	Alteration of endosomal trafficking	40%	[95,96,97]

SQSTM1/p62 has originally been linked to neurodegeneration due to its presence in ubiquitin-positive cytoplasmic aggregates of misfolded proteins, and p62 knockout mice show brain and behavioral changes compatible with neurodegeneration. A number of studies have reported an expanding list of heterogeneous SQSTM1/p62 mutations, localized to several different regions essential for protein function, in patients with both familial and sporadic ALS/FTLD; moreover, missense and truncating mutations in the same protein are thought to be causative of the chronic progressive skeletal disorder Paget’s disease of the bone (PDB). Thus, mutations of the *SQSTM1* gene are associated with different disease phenotype, and the same *SQSTM1* variant has been found to be shared by PDB and ALS patients within the same family (reviewed in [32,34]).

- Dominant missense, recessive deletions and nonsense mutations of optineurin have been identified in both patients with FALS and SALS [87]. The relevance of optineurin mutations to ALS include impairment of the recruitment of damaged mitochondria to autophagosomes, as well as sequestration of functional proteins in hybrid complexes, which compromises the maturation of autophagosomes [35,56]. Furthermore, TDP-43- or SOD1-positive inclusions of sporadic and SOD1 cases of ALS have been found to contain also optineurin [87]. Interestingly, as in the case of p62, mutations of optineurin are also associated with PDB and, in addition, with glaucoma [33,34].
- Mutations affecting the *UBQLN2* gene were originally found as a cause of ALS by Deng *et al*. in 2011. Interestingly, ubiquitin pathology was not restricted to patients with ALS who had ubiquilin-2 mutations, but also to SALS and ALS cases with other mutations, in which UBQLN-2-positive inclusions could be identified in the brain and spinal cord [89]. Moreover, ubiquilin-2 (UBQLN2) binds with high affinity to TDP-43 and modulates TDP-43 levels. Given the presence of ubiquilin-2 in inclusions containing also other proteins (such as TDP43), UBQL2 has been suggested to play a role in ALS that may not be uniquely associated with its mutations [75]. Interestingly, a link between ALS-linked ubiquilin-2 and optineurin mutations has been recently proposed, since they both appear to interfere with the trafficking of endosomal vesicles, suggesting that these proteins may function in a common pathological process [38].
- Mutations in the valosin-containing protein VCP/p97 lead to a multisystem degenerative disease consisting of inclusion body myopathy, early-onset Paget’s disease of the bone and fronto-temporal dementia (IBMPFD) [98]. Subsequently, mutations in *VCP* were found in a small percentage of familial ALS cases [90]. As discussed in the first section of this review, VCP is essential for ubiquitin-containing autophagosome maturation and endolysosomal sorting, as well as for establishing the fusion of lysosomes with autophagosomes; in addition, the expression of either disease-associated VCP mutants or dominant-negative VCP results in autophagy defects ([55] and the references therein). Very interestingly, VCP appears to play an important role in the autophagy-mediated degradation of stress granules, thus contributing to the formation of stress granule-based aggregates, a process that may indirectly impact RNA processing [42]. This function links VCP to two genes more commonly associated with FALS, which code for the RNA binding proteins TDP-43 and FUS (reviewed in [34,75]). An emerging idea is that the changes in RNA processing observed in ALS-FLTD may be the result of pathological stress granule formation, normally requiring intact autophagy, lysosomes and VCP [99]. Further, other ALS-FTLD-associated gene products, such as profilin 1 and ataxin 2, are stress granule-associated proteins [100]. Finally, *VCP* mutations have also been shown to affect the nutrient sensing function of mTOR, the main regulator of starvation-induced autophagy, although the precise mechanism remains unclear (reviewed in [11]).
- Another ALS-causing gene with a clear function in autophagy is dynactin 1 (DCTN1) [91]. Mutations that affect the dynein motor machinery impair autophagosome-lysosome fusion, leading to decreased autophagic clearance of aggregated proteins and enhanced toxicity, while motor neuron-specific knockdown of the dynactin 1 homologue in the nematode was shown to disrupt autophagosome transport and induce motor neuron degeneration ([55,101] and the references therein).
- A rare mutation in the endosomal ESCRTIII-complex subunit CHMP2B, which, as mentioned before, is part of the ESCRT-complex machinery whose activity is required to maintain autophagic flux, was found in human patients with an autosomal dominant form of FTLD linked to chromosome 3. Additional alterations have been reported in association with ALS phenotypes, thus strengthening the idea of a common molecular pathology between ALS and FTLD [47,92].
- The convergence of the autophagic pathway with the endocytic pathway is underlined by the finding that the polyphosphoinositide phosphatase-encoding gene *FIG4* (also known as *SAC3*) is mutated in some cases of FALS [93]. Given the importance of its enzyme product in the regulation of the intracellular levels of phosphatidylinositol 3,5-biphosphate and its metabolite phosphatidylinositol 3 phosphate (PI3P), and, consequently, in the lipid composition of late endosomal/lysosomal membranes, mutant *FIG4* is thought to interfere with the trafficking of these organelles and, presumably, with a proper autophagic function, thus implicating phosphoinositide metabolism in the pathogenesis of ALS [74,75]. Mutations in this gene are also responsible for a variant of the Charcot-Marie-Tooth disorder (CMT4), characterized by axonal degeneration [50].
- Mutations in the *ALS2* gene, which encodes the protein alsin, account for several recessive MND, including ALS2. Alsin is a guanine nucleotide exchange factor (GEF) for the small GTPase protein Rab5 and, through its activation, is involved in endosome fusion and trafficking, as well as neurite outgrowth [94], reviewed in [74]. More recently, pathogenic missense mutations of alsin were shown to impair activation of Rab5, resulting in disturbances in the formation of amphisomes in cultures cell models [102]; reviewed in [55].
- It is noteworthy that the protein C9ORF72, encoded by the most commonly-mutated gene in ALS, has been recently found to be structurally related to a GDP/GTP exchange factor (GEF) that activates Rab-GTPases [103] and to co-localize with Rab proteins, including Rab7, in neuronal cell lines, primary cortical neurons and human spinal cord motor neurons of ALS patients [97]. Since the small GTPase Rab7 is needed for the maturation of autophagosomes to autolysosomes (Figure 1), these studies on the still undeciphered function of C9ORF72 suggest that, unexpectedly, it may play a role in endocytic trafficking and autophagy (reviewed in [5,77,81]).
- Finally, to complete this overview, it should be reported that the loss of function of some ALS-linked proteins that are neither bona fide autophagy receptors/adaptors nor directly involved in the process of autophagy, can be associated with defective protein degradation. For example, a mutation in the transmembrane domain of the sigma receptor-1 protein (SigR1), an ER chaperone protein located at the mitochondria-ER interface, has been found to cause a form of juvenile ALS and FTLD [104,105], and more recently, depletion of SigR1 has been shown to be associated with defects in endosomal trafficking and accumulation of autophagic substrates [106]. Although this protein was originally described as a sigma-opioid receptor, it was later found to be a distinct non-opioid receptor that binds a wide range of ligands and with a crucial role in Ca^++^ signaling, neuronal function and survival [105,107].

Despite the increasing evidence that altered proteostasis is crucially involved in the pathogenesis of ALS, the effects of the above-described mutations in an *in vivo* cell-specific context remain to be elucidated. Since autophagy receptor proteins are involved in the regulation of a variety of physiological processes, dissecting the precise effects of their disease-associated mutations is often a challenging task, and the role of autophagy receptor genes mutations in ALS pathogenesis is still under investigation. As previously pointed out [75], the presence of mutations affecting proteins that normally contribute to efficient degradative pathways, such as autophagy, does not necessarily indicate a deficit in protein turnover as the pathogenic mechanism. Not only may these proteins have additional functions with a more important role in the disease mechanism, their physiological role may also vary among different cells types according to their specific interactions within a given cell population. For example, in neurons, a direct interaction has been described between VCP/p97 and neurofibromin, the protein product of the neurofibromatosis type I (*NF1*) gene [108], which appears to be essential for proper dendritic spinogenesis and synaptogenesis. Hence, dysfunction of VCP/97 due to a mutation in its gene may result in neurodegeneration because of its altered contribution to neuronal morphogenesis [109]. Moreover, whether the proteasomal system or the autophagic machinery is at the heart of the degradation failure in ALS is still a matter of debate, due to the intense crosstalk between the two systems. Notably, evidence obtained in *in vivo* experimental models has shown that transgenic mice with motor neuron-specific knockout of proteasomes, but not of autophagy, develop ALS phonotypes, suggesting that a cell-specific sensitivity may exist to either proteasomal or autophagic failure [110]. Thus, although the striking correlations between mutations in autophagy-related genes and ALS pathology suggest that altered autophagy is a crucial component underlying neuronal loss in MND, additional studies will be necessary to conclusively characterize the pathogenic significance of these mutations and to directly demonstrate their relevance to motor neuron-specific degeneration. In this regard, in apparent self-contradiction and in contrast with the view that dysproteostasis caused by malfunction of degradation machinery contributes to motor neuron failure in ALS, studies from our laboratory on a mutant form of the *VAPB* gene product, associated with a rare form of FALS, indicate that mutant VAPB generates unusual inclusions, which are easily cleared by cells and do not affect either UPS or autophagy. Below, we recapitulate and discuss our recent findings.

## 4. VAPB

VAPB, and its homologue VAPA, are members of the highly-conserved and ubiquitously-expressed VAP (vesicle-associated membrane protein (VAMP)-associated protein) family of ER tail-anchored transmembrane proteins. VAPA and VAPB share high (63%) sequence identity and similar domain organization, with a cytosolic N-terminal domain, highly homologous to the nematode major sperm protein (MSP), a central coiled-coil domain and a C-terminal transmembrane domain bearing a putative dimerization motif, which allows VAP proteins to undergo homo- and hetero-dimerization [111]. Oligomerization appears to be independent of the MSP domain [112].

VAP proteins function as receptors for cytosolic proteins to recruit them to the surface of the ER. The interaction between these proteins and VAP is mediated by the so-called FFAT motif (two phenylalanines in an acidic tract) in the ligands and a binding pocket in the MSP domain of the VAPs (reviewed in [111]). Due to the wide spectrum of their interacting proteins, VAPs have been shown to be implicated in a great variety of physiological functions, ranging from membrane trafficking [113,114], interorganellar lipid exchange ([115], reviewed in [111]) and Ca^++^ homeostasis [116] to cytoskeleton organization [117], synaptic morphology and physiology, neurite extension [118,119,120] and the unfolded protein response (UPR) [121]. Importantly, the VAPs have been implicated in the genesis of membrane contact sites between the ER and other organelles, including the plasma membrane, endosomes, the yeast vacuole and the trans-Golgi network [115,116,122,123,124,125,126]. A similar VAP-mediated ER-recruitment mechanism has been shown to lie at the basis of the formation of MCSs between the ER and membrane-bound intracellular inclusions driven by and hosting human infectious agents, such as *Chlamydia trachomatis* [127].

The precise role of VAP proteins in the regulation of the UPR has not been conclusively defined; however, it is thought that they act as stress sensors, facilitating activation of the UPR by enhancing the splicing of XBP1 via the classical transmembrane kinase Ire1 [128]. In contrast to this observation, another study [129] reported that VAPs negatively modulate the activity of the UPR-related transcription factor ATF6 (reviewed in [121]).

Although VAP proteins have been implicated in many processes, perhaps the best documented function and the one most relevant to neurodegeneration is its role in recruiting lipid exchange proteins to the ER membrane [115]. This depends on their ability to interact with the FFAT motif of ER lipid sensing, binding (such as oxysterol binding protein (OSBP) and OSBP-related proteins (ORPs)) or transfer (such as ceramide transport protein (CERT) and phosphatidylcholine-phosphatidylinositol exchange protein Nir2) proteins, thus mediating the non-vesicular transport of sterol, ceramide and phospholipids occurring at ER-Golgi and ER-plasma membrane contact sites [115,130,131]. These contacts, in turn, are the sites for regulation of phosphoinositide levels, such as that phosphatidylinositol 4-phosphate-PI(4)P, a crucial molecule for the maintenance of organelle identity, regulatory functions and membrane trafficking. Lipid traffic between the ER and the Golgi mediated by lipid exchange proteins is tightly linked to the turnover of PI(4)P, present at different concentrations on both partner membranes, and its regulation involves the PI(4)P dephosphorylating enzyme Sac1, which also binds VAP proteins via their transmembrane domain [132,133]. Silencing of the VAPs has been shown to affect the lipid composition of the Golgi complex and to have adverse effects on membrane traffic between the ER and the Golgi [115].

In addition to the strong FFAT motifs already known, there are additional proteins with weaker non-canonical FFAT motifs, which may be functionally important VAP interactors and include, for example, proteins involved in ER stress and the protein FAF1, an ubiquitin-binding cofactor for the p97 ATPase [134,135]. These findings may suggest a FAF1-mediated interaction between VAPs and p97, thus adding a level of complexity for VAPs function and possibly implicating them in p97 ATPase-regulated processes. Along this line and relevant to this review, VAPB has been reported to bind Rab3GAP1, the catalytic subunit of the Rab3 GTPase-activating protein complex. This protein plays an important role in the coordination of cellular vesicle transport, and in addition to regulating neurotransmitter release at the neuronal synapse, has been recently shown to affect protein aggregation and influence autophagosomal biogenesis both in basal and stimulated autophagy, as well as to be involved in nuclear envelope formation [136,137].

Lastly, studies in *Drosophila* and *C. elegans* have shown that the MSP domain of VAP proteins can be cleaved from the transmembrane domain and may function as a secreted ligand by binding to ephrin receptors and other axon guidance receptors to stabilize mitochondria networks at post-synaptic neuromuscular junctions. However, the mechanisms of the processing and of the secretion of the cytosolic VAP domains have not been addressed [138,139].

Given the wide and increasing spectrum of VAPA and VAPB interactions, these proteins may be considered as major platforms for integrating many inputs into the ER. This makes it difficult to predict the physiological significance of the loss of VAP, for example in motor neuron disease. It must be noted that, up till now, specific roles that functionally distinguish the two VAP isoforms have not been identified.

## 5. Role of the *VAPB* Gene in the Pathogenesis of ALS

The discovery that the *VAPB* locus is linked to ALS heightened interest in the VAP proteins and in the disease mechanism of the mutant forms. A missense mutation in the *VAPB* gene was identified at locus 20q13.3, which consists of the substitution of a highly conserved proline residue at position 56 with a serine (P56S) in the MSP domain of the VAPB protein [140]. The mutation is associated with three autosomal dominant human MND: a late-onset spinal muscular atrophy (SMA), an atypical ALS type 8 (ALS8) and a typical severe ALS with rapid progression [140,141]. Initially identified in eight Brazilian families with a shared Portuguese ancestor, the same mutation was subsequently detected in an unrelated German patient, with a haplotype distinct from the one linked to the mutation in the Brazilian families [142]. Subsequently, three other mutations, one of which is in the MSP domain (T46I) [143] and another in the transmembrane domain (V234I) [144,145] of VAPB, have been identified [146]. Pathogenicity of *VAPB* mutations has been conclusively shown only for the P56S mutation, which is known to affect the folding properties of the MSP domain *in vitro*, leading to loss of its native structure and exposure of hydrophobic patches, with the consequent facilitation of protein aggregation [112,147,148]. Although the ALS-linked *VAPB* gene is rare and one of the least understood, the observation that VAP levels are decreased in sporadic ALS patients and animal models in which the VAPB gene is not mutated (such as mutant SOD1 mice) [147,149] is consistent with a general role of the VAPs in the pathogenesis of both SALS and FALS and suggests that elucidation of the cellular effects of the mutant gene will bring important insights into the molecular pathogenesis of ALS.

Like other proteins linked to neurodegenerative diseases, mutant VAPB, transiently expressed in a variety of cultured cells, forms intracellular inclusions; for this reason, mutant VAPB-associated ALS has long been regarded as a protein misfolding disorder [116,147,150,151,152], implying that the efficiency of the removal of the mutant protein from the cell may be an important factor in its toxicity.

Many hypotheses that have been proposed for mutant P56S-VAPB proteotoxicity are based on the interaction of VAPB with its many different protein partners, including VAPA. On the one hand, it has been observed that P56S-VAPB intracellular inclusions sequester both the wild-type protein and, to a lesser extent, VAPA [118,128,147,153,154]. This suggests that the dominant inheritance of ALS8 is due to a loss of function resulting from a dominant negative effect of mutant VAPB protein. On the other hand, cellular alteration can derive from sequestration of functionally important VAPB interactors. For example, sequestration of the ER-Golgi recycling protein Yif1A, located in the ER-Golgi intermediate compartment (ERGIC), within VAPB inclusions [114], may underlie the observed impairment of the secretory pathway in P56S-VAPB overexpressing cells [115]. Since both VAPB and YIF1A are required for membrane trafficking to dendrites and proper dendrite morphology, this mechanism may be crucial for the development of motor neuron disease [114]. Similarly, the pathological recruitment of phosphoinositide phosphatase Sac1, due to its interaction with endogenous VAPB, in P56S-VAPB inclusions, could lead to abnormally elevated and disruptive levels of phosphoinositides, as described in Drosophila motor neurons [133]. Another contribution to mutant VAPB pathogenicity, possibly as a consequence of its physical interaction with wt-VAPB or its direct binding to activating transcription factor 6 (ATF6), could derive from its reported role in the UPR, since overexpression of P56S-VAPB has been reported both to attenuate UPR signaling [128] and to increase ER stress in animal disease models [129,138,155,156]. Given the existing crosstalk between ER stress, UPS and autophagy, these alterations could impact motor neuron viability. Finally, according to a recent genetic screen performed in *Drosophila*, both the wt- and mutant ALS-linked VAPB interact with *TOR* (target of rapamycin), the important regulator of cellular anabolic and catabolic functions, as well as potent repressor of autophagy, suggesting that P56S-VAPB could be involved in disturbances of several aspects of the proteostasis network [157].

**Figure 2:**
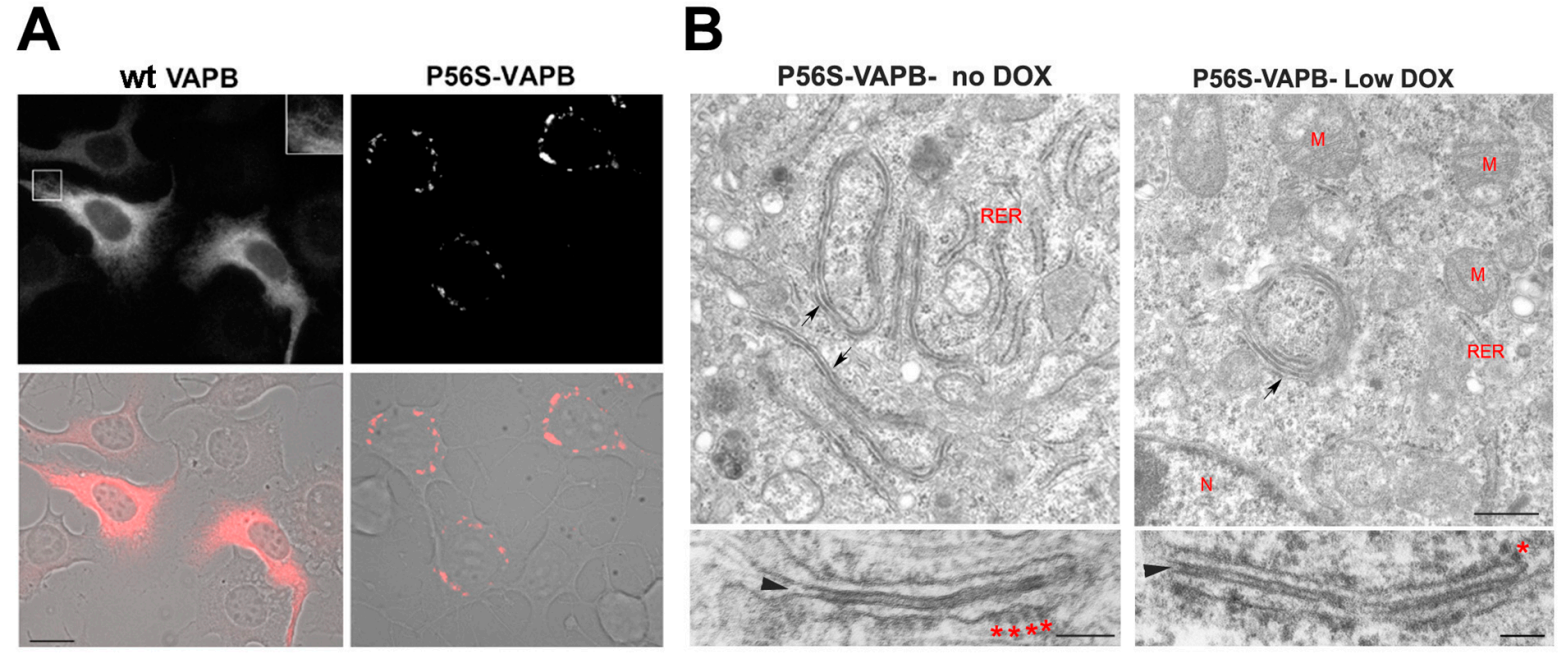
Mutant VAPB inclusions in a model motor neuronal cell line (NSC34 cells) (**A**) and in HeLa cells (**B**). (**A**) NSC34 cells stably transfected with wild-type (left) or P56S-VAPB (right) under a Tet-repressible promoter were grown in the absence of the antibiotic to induce expression of the transgene. Shown are fluorescence images (top) and fluorescence superimposed on the phase contrast images of the same two fields (bottom). The inset of the upper left panel shows a two-fold enlargement of the boxed area and illustrates the web-like distribution of wt VAPB, typical of an ER protein, as opposed to the clusters of mutant protein that congregate around the nucleus. Modified from [160]. Scale bar, 15 µm. (**B**) HeLa cell lines, expressing P56S-VAPB under a Tet-repressible promoter, were grown in the absence of the antibiotic (left) or at low concentration (right, 0.1 ng/mL) and then analyzed by transmission EM. Arrows in the upper panels indicate the paired cisternae with interposed dense cytosolic layer; such structures can be visualized both in moderately (left) and low (right) expressing cells. The lower panels show the inclusions at higher magnification. The arrowheads and asterisks indicate the dense cytosolic layer and attached ribosomes, respectively. RER, rough endoplasmic reticulum, M, mitochondria, N, nucleus. Scale bars: upper panels, 500 nm; lower panels 100 nm. Modified from [159].

A caveat to the experimental basis of all these hypotheses is that most of the studies were performed on cultured cells acutely overexpressing mutant VAPB, which may not be adequate models to unravel the effects of P56S-VAPB, as they do not clearly mimic the situation in cells chronically expressing the mutant protein from a single allele, as in patients’ cells. To bypass this limitation, we generated both non-neuronal (HeLa) and motor neuronal (NSC34) cell lines inducibly expressing mutant VAPB under the control of a Tet-repressible promoter. In doxycycline-free medium, these cells express P56S-VAPB chronically at moderate levels, comparable to those of the endogenous protein, and form cytoplasmic inclusions (Figure 2) [158,159,160].

Using these cell lines, our studies revealed important differences between P56S-VAPB inclusions and other inclusion bodies linked to neurodegenerative disorders. In particular, mutant VAPB inclusions correspond to a profoundly restructured ER domain and not to a cytosolic protein aggregate, as is generally the case. Our work [158] demonstrated that P56S-VAPB, like the wild-type (wt) protein, inserts into the ER membrane via its C-terminal tail, but that after insertion, it rapidly clusters, generating tightly-apposed ER cisternae apparently held together by the interactions between the mutant cytosolic domains. Indeed, immuno-EM analysis demonstrated the presence of P56S-VAPB exclusively in the dense cytosolic layer separating the paired cisternae [158]. The restructured ER induced by P56S-VAPB differs from typical organized smooth ER, observed upon overexpression of dimerizing ER membrane proteins (OSER; [161]). Strikingly, however, it resembles the stacked ER cisternae induced by the IP3 receptor in Purkinje neurons [162].

We also demonstrated that the inclusions contain polyubiquitylated P56S-VAPB and are localized in an iuxta-nuclear position in close association with the Golgi complex, although, at variance with “classical” protein aggregates, they do not require microtubules to reach this position [159]. Our most surprising observation was that mutant VAPB is efficiently extracted from the ER and degraded by the proteasome more rapidly than the wt protein, both in HeLa and NSC34 cells [159,160]. This behavior distinguishes VAPB inclusions from the “classical” aggregates of neurodegenerative diseases.

Proteasomal degradation of P56S-VAPB requires the AAA ATPase p97 and leads to efficient clearing of the inclusions by the UPS pathway, and not by autophagy [160]. Moreover, when we compared the distribution of mutant VAPB with that of the ubiquitin-binding autophagy receptor p62, we found no co-localization between the two proteins, in striking contrast with the observations reported for inclusion bodies of other diseases [159]. Although TDP-43 has been found to be a major component of ubiquitylated, p62-positive protein aggregates in ALS patients (see the previous paragraphs), we failed to detect any redistribution from the nucleus to the cytoplasm or coincident localization of the latter protein with mutant VAPB inclusions [159]. Notwithstanding their exclusive clearance by the proteasome under basal conditions, with no apparent involvement of autophagy, in neuronal and non-neuronal cells, P56S-VAPB inclusions become an autophagosomal substrate under conditions that activate non-selective autophagy, such as starvation [160]. The fact that P56S-VAPB degradation can be enhanced by autophagy when this is stimulated is in line with the concept that autophagy can provide a support system for the UPS to clear ubiquitylated substrates. Interestingly, we found that the levels of endogenous wild-type VAPB, which is partially recruited to mutant VAPB inclusions [147], were not affected by the presence of the rapidly-degraded inclusions [159]. Our observations may explain why VAPB inclusions have not been observed in motor neurons derived from induced pluripotent stem cells of patients carrying the P56S-VAPB mutation [163] and suggest that the slow onset of P56S-linked familial ALS is not a consequence of the progressive accumulation of the mutant protein over time.

Since, as highlighted in the previous pages of this review, a substantial body of evidence indicates that disturbances of proteostasis represent an important mechanism of proteotoxicity of pathogenic aggregates in neurodegeneration, we investigated a possible interference of P56S-VAPB, expressed at moderate levels, with autophagic flux and the UPS. Indeed, overexpressed mutant VAPB has been reported to interfere with proteasomal function [164]. In contrast to our expectations, in our inducible HeLa TetOff cells, neither proteasome-mediated degradation nor autophagic flux are altered by P56S-VAPB inclusions. In particular, clearance of a classical ERAD substrate, whose degradative pathway shares with the one of P56S-VAPB the involvement both of the proteasome and of p97, was unaffected by the expression of the mutant protein. Likewise, when autophagy was stimulated either pharmacologically or by starvation, the behavior of the two autophagosomal markers p62 and LC3 was found not to be affected by P56S-VAPB expression. Thus, it appears that cells can adjust the capacity of their degradative machinery to cope with moderate levels of mutant VAPB without consequent disturbances in proteostasis [160].

A second fundamental process, in which the VAPs are implicated, is intracellular transport through the secretory pathway, but contrasting results have been reported on the effect of P56S-VAPB expression on intracellular transport. As a matter of fact, both a strong interference of overexpressed P56S-VAPB (and also of overexpressed wt VAPA) with VSVG transport [165,166] and no delay of the transport of the same secretory membrane cargo in primary hippocampal neuronal cultures have been detected [147]. In our system, we found that neither transport from the ER to the Golgi nor export to the cell surface of a model secretory membrane protein were altered by the presence of P56S-VAPB inclusions. We conclude that cells can maintain secretory pathway function in the presence of P56S-VAPB inclusions, notwithstanding their close relationship to the Golgi apparatus [160]. Hence, not only do our results reveal surprisingly efficient extraction from the ER and proteasomal degradation of a severely aggregated mutant protein, but they also demonstrate a lack of interference of cytoplasmic inclusions expressed at moderate levels with either proteasome-mediated degradation, basal autophagic flux or secretory pathway function.

The lack of interference of mutant VAPB with basic cellular processes raises the question of whether a toxic gain-of-function of mutant VAPB contributes in any way to the process of neurodegeneration. We speculate instead that the ALS8 phenotype may result from haploinsufficiency. Consistent with this idea, one study on P56S-VAPB transgenic mice (which produce normal amounts of the wild-type protein) failed to reveal the development of any motor deficit [167]. Analysis of another transgenic animal model revealed that development of mild motor abnormalities and loss of cortical, but not spinal, motor neurons, requires the expression of very high levels mutant VAPB [155]. Other transgenic mice strains, although presenting P56S-VAPB-containing inclusions in motor neurons, showed no motor abnormalities [114,168].

The above-described negative results might be explained by the use of mouse models in which expression of mutant VAPB was restricted to CNS neurons. Indeed, it is feasible that P56S-VAPB-driven development of ALS is non-cell autonomous, as is the case for ALS-linked mutant SOD1 [79]. However, given the lack of clear evidence for P56S-VAPB toxicity, we believe that the haploinsufficiency hypothesis deserves further investigation. In this respect, it is interesting that VAPB depletion was found to cause alterations in muscle lipid metabolism [169] and that VAPB-deleted mice, although free from a complete ALS phenotype, developed mild motor deficits after 18 months of age [146].

If an insufficient level of functional VAPB is the primary cause of motor neuronal degeneration in ALS8, the question arises as to whether VAPB plays a particular role in motor neurons not performable by VAPA, or whether, more simply, motor neurons require the full gene dosage of VAPA + B for long-term health and survival. Because of the multiple roles of the VAP proteins, loss of 50% of functional VAPB could have a wide spectrum of small effects, which would be difficult to pinpoint in cultured cell and animal models, but which could lead to a slow build-up of damage with cell death as the final outcome. Given the reported decrease in both VAPA and B in SALS patients and in SOD1 transgenic mice, the roles of both VAP isoforms in motor neurons will surely be the subject of intense investigation in the coming years.

## 6. Concluding Remarks

In this review, we have summarized evidence in favor of the importance of autophagy in aggregate-prone protein clearance and its crucial contribution to the initiation of neurodegenerative diseases, such as ALS. Nevertheless, it is not always clear at what point of the neurodegenerative cascade proteostasis failure steps in, and it is likely that the sequence of events is different in different types of ALS. Indeed, although the hallmark of all MNDs is motor neuronal death, there are presumably many different initial triggering events, with different pathways converging on the final fatal outcome.

The work from our laboratory summarized here indicates that the mutant form of VAPB responsible for a dominantly inherited form of ALS is not toxic and suggests that the disease mechanism may be principally due to haploinsufficiency. An important goal will be to establish whether the reduced gene dosage of VAPB in motor neurons impinges on autophagy, thus linking this form of FALS to others, in which perturbation of proteostasis has been demonstrated.
